# Epicardial vasospasm and concomitant ventricular tachycardia treated with Beta-1-specific Beta-blockade: a case series in support of nebivolol

**DOI:** 10.1093/ehjcr/ytaf641

**Published:** 2025-12-16

**Authors:** Sarah A Miner, Laurie-Anne Boivin-Proulx, Mary McCarthy, Lynne E Nield, Mouhannad M Sadek, Steven E S Miner

**Affiliations:** Department of Anatomy and Cell Biology, McGill University, 3640 Rue University, Montreal, QC H3A 2A7, Canada; Cardiac Program, Southlake Regional Health Centre, 596 Davis Drive, Newmarket, ON L3Y 2P9, Canada; Department of Cardiology, University of Ottawa Heart Institute, 40 Ruskin Street, Ottawa, ON K1Y 4W7, Canada; Cardiac Program, Southlake Regional Health Centre, 596 Davis Drive, Newmarket, ON L3Y 2P9, Canada; Women and Babies Program, Sunnybrook Health Sciences Centre, 2075 Bayview Ave, North York, ON M4N 3M, Canada; Cardiac Program, Southlake Regional Health Centre, 596 Davis Drive, Newmarket, ON L3Y 2P9, Canada; Department of Medicine, University of Toronto, 27 King’s College Circle, Toronto, ON M5S 1A1, Canada; Cardiac Program, Southlake Regional Health Centre, 596 Davis Drive, Newmarket, ON L3Y 2P9, Canada; Department of Medicine, University of Toronto, 27 King’s College Circle, Toronto, ON M5S 1A1, Canada; School of Kinesiology and Health Science, York University, 170 Campus Walk, Toronto, ON M3J 1P3, Canada

**Keywords:** Case Report, Epicardial Vasospasm, Ventricular Arrhythmia, Ventricular Tachycardia, Cardiac Arrest, Nebivolol

## Abstract

**Background:**

Patients who experience ventricular tachycardia and cardiac arrest induced by epicardial vasospasm are at high risk for recurrent cardiac events. Conventional treatment includes calcium channel-blockade, long-acting nitrates, and the withdrawal of beta-blockade. These guidelines have not been proven effective in randomized controlled trials, and the evidence against beta-blockade is primarily anecdotal. Ongoing medical management in the setting of treatment failure is unclear, but abnormal sympathetic activity has been implicated in both spasm and ventricular arrhythmias.

**Case presentation:**

We describe three patients with spasm-related ventricular arrhythmias and unacceptably poor response to conventional treatment. Clinical stability and asymptomatic status were achieved following the addition of nebivolol, a third-generation, lipophilic beta-1-specific beta-blocker.

**Discussion:**

Selective beta-blockade may represent a therapeutic option in patients with high-risk epicardial spasm and ventricular arrhythmias. Furthermore, the apparent success of nebivolol in this setting suggests that hyperactive sympathetic input may represent a causal or aggravating factor in spasm-associated ventricular arrhythmias.

Learning Points

- Nebivolol, in combination with other vasodilatory therapy, may be beneficial in high-risk epicardial vasospasm with concomitant ventricular tachycardia patients.

+

- The role of adrenergic input should be considered in patients with concomitant vasospasm and ventricular tachycardia.

## Introduction

Epicardial vasospasm is a form of vasomotor coronary artery disease involving the acute, transient narrowing of a coronary artery.^1–3^ It is a common cause of angina, myocardial ischaemia, and myocardial infarction, with complex underlying pathophysiology.^1–3^ Hyperreactivity of coronary vascular smooth muscle cells (VSMCs) to vasoconstrictors is suspected to play an important pathophysiological role.^1–3^ Current guideline-directed therapy includes long-acting nitrates (LANs), which increase the supply of nitric oxide, and calcium channel blockers (CCBs), which reduce VSMC reactivity.^1–3^ Prognosis is generally good,^4^ but those whose spasm precipitates ventricular arrhythmias and cardiac arrest are subject to a 38% event rate at 5-year follow-up with a hazard ratio of 3.25 compared to vasospasm patients without cardiac arrest.^5^

Beta-blockers represent the first-line therapy for cardiac arrest for their anti-ischaemic and antiarrhythmic effects,^6^ but are contraindicated in the setting of vasospasm^3^ due to risk of unopposed alpha-adrenergic receptor-mediated vasoconstriction, resulting in exacerbated anginal symptoms.^7^ Consequently, patients with both vasospasm and ventricular tachycardia (VT) are typically prescribed CCBs and LANs, but treatment failure is common.^5^ This problem is further complicated by registry data suggesting that chronic nitrate therapy may introduce an increased risk of acute coronary syndrome in vasospasm patients.^8^ These poor clinical outcomes existing in the setting of standard therapy imply a need to reconsider the conventional approach.

Nebivolol, a third-generation lipophilic beta-blocker, is highly cardioselective to beta-1 adrenergic receptors, leaving beta-2- and -3-mediated vasodilation intact and thus evading the conventional concern of beta-blockade.^9^ While additionally promoting the production and bioactivity of nitric oxide,^10^ nebivolol may have a potential role in the management of high-risk vasospasm.

In this observational and hypothesis-generating study, we present three patients with vasospasm and VT who were unresponsive to CCBs and/or LANs and then achieved clinical stability with nebivolol treatment.

## Summary figure

**Figure:**
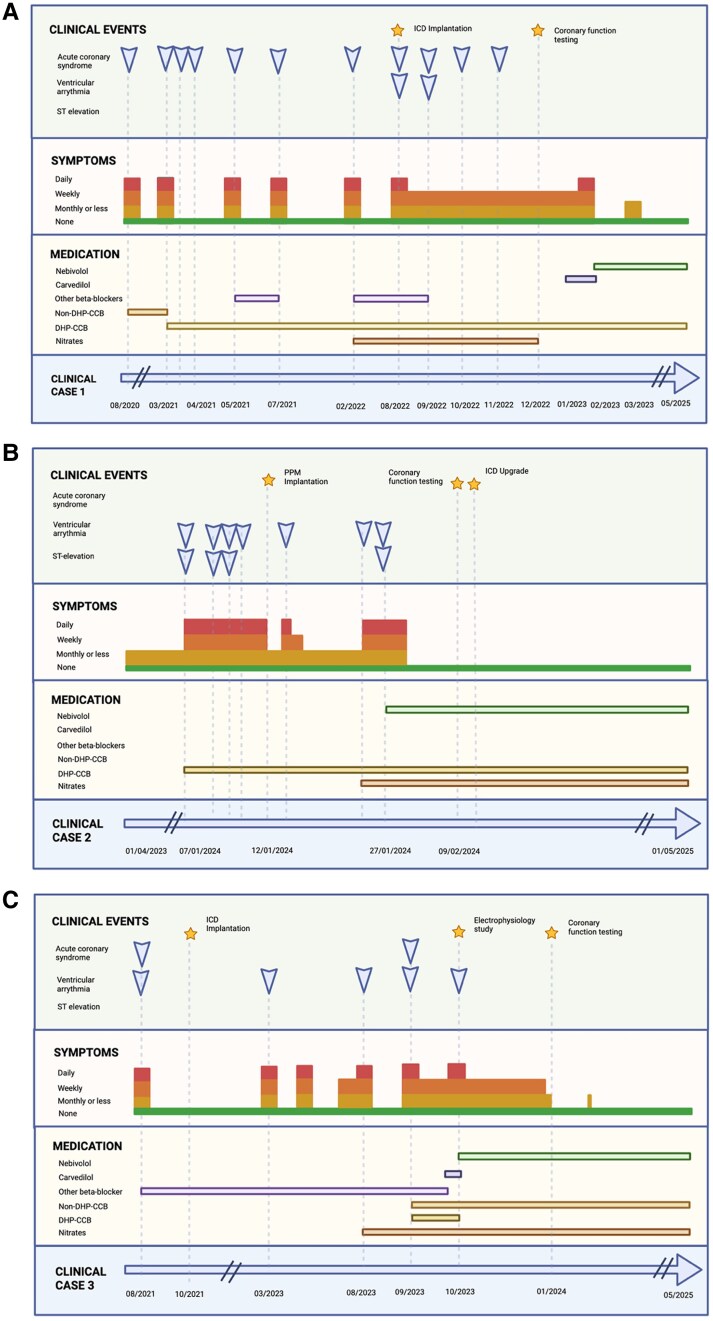
Arrows represent the incidence of the respective clinical event. Stars represent the occurrence of the respective testing or surgical event. Symptom severity is depicted by height and colour, with the severity increasing as height does and as the colour transitions from green to red. Active medications are represented temporally by coloured bars; for example, Patient 1 was on nitrates from 02/2022 until 12/2022. Dates are formatted as DD/MM/YYYY and MM/YYYY. ICRT, Invasive Coronary Reactivity Testing; DHP-CCB, Dihydropyridine Calcium Channel Blocker; ICD, Implantable Cardioverter-Defibrillator. Made with BioRender.

## Case presentations

**Figure 1:**
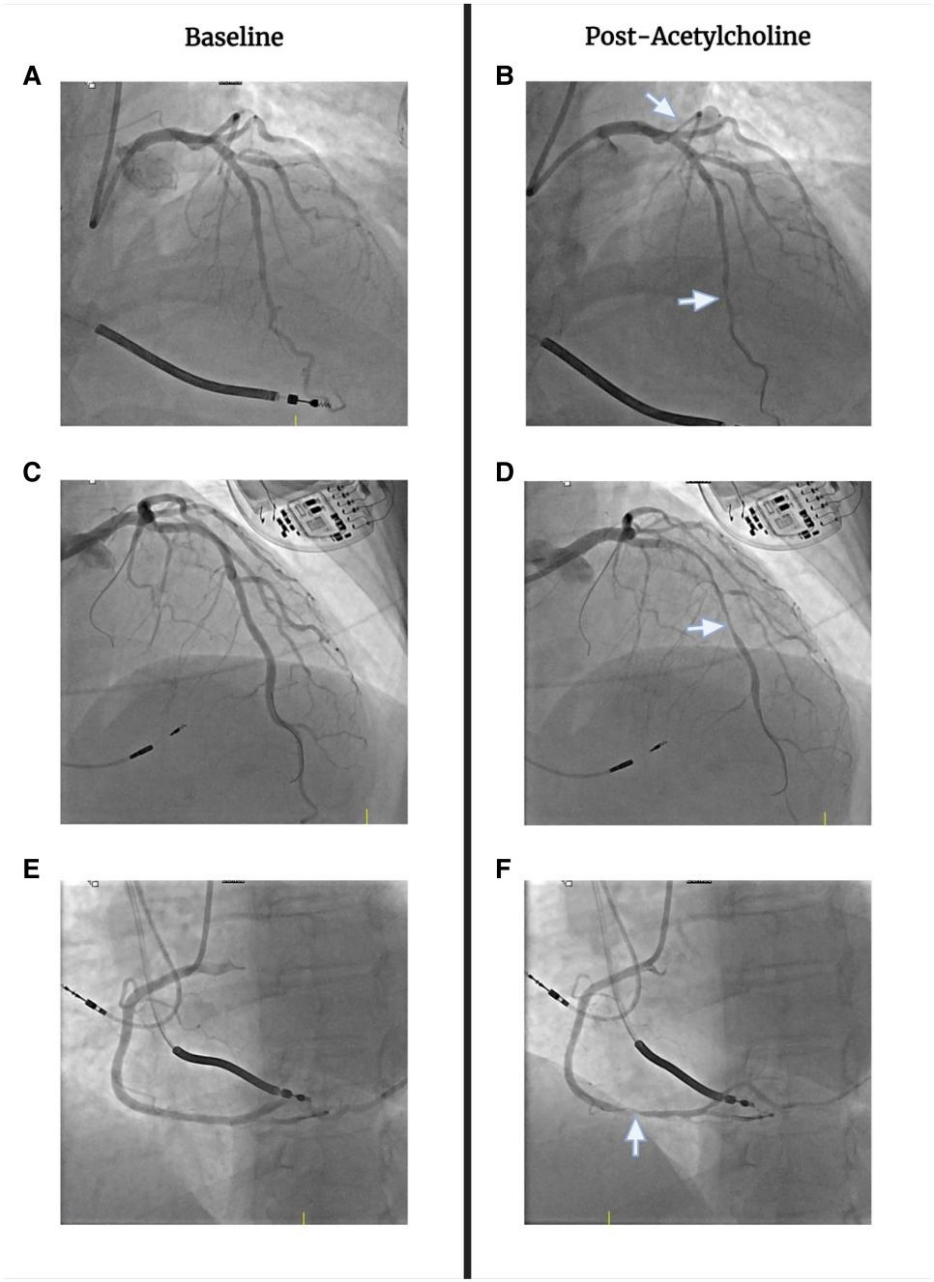
A & B = patient 1; C & D = patient 2; E & F = patient 3. Arrows point to spasm. Made with BioRender.

*Figure 1* exhibits clinical timelines. *Figure 2* displays invasive coronary reactivity testing (ICRT) images. Supplementary material online, *Figures* include relevant electrocardiograms and telemetry.

### Patient 1

A 48-year-old female presented on 08/2020 to a quaternary care centre with myocardial infarction with non-obstructive coronary arteries (MINOCA). Cumulatively through 11/2022, she suffered eleven MINOCA, with ‘coronary vasospasm’ being the working diagnosis throughout. She was treated initially with diltiazem but switched to nifedipine (60 mg PO daily) and nitropatch (0.6 mg transdermal daily). She consistently presented after chest pain had resolved with troponin-T-Hs generally between 42–97 ug/mL and electrocardiograms revealing anterior T-wave inversions.

She presented on 08/2022 with cardiac arrest following chest pain, resulting in four attempted defibrillations for ventricular fibrillation before successful cardioversion to sinus rhythm. Severe anterior hypokinesis, 488 ug/mL troponin-T-Hs peak and right bundle branch block were noted. She received an implantable cardioverter-defibrillator (ICD).

Cardiac magnetic resonance images (MRI) revealed an inferior scar consistent with infarction. Positron emission tomography scan revealed mildly reduced myocardial flow reserve. Coronary angiography revealed 30–40% stenosis of the left anterior descending (LAD) artery. Despite ongoing nifedipine and nitropatch therapy, the patient continued to report significant anginal burden.

She underwent ICRT 12/2022 at a tertiary care centre. As per protocol, vasoactive medications were withheld for two days, which prompted unstable symptoms requiring hospital admission. Microvascular function was normal. Following the intracoronary acetylcholine test dose (20ug), she developed multifocal spasm and ST-elevation. LANs were permanently discontinued. Treatment was initiated with carvedilol (6.25 mg BID) (nebivolol not on formulary), amlodipine (2.5 mg PO daily), and rosuvastatin (40 mg PO daily). She had no further anginal episodes. She was discharged on these medications, except with nebivolol (5 mg PO daily) replacing carvedilol. Apart from one episode of chest pain precipitated by anxiety within the first three months, she has been pain-free and event-free over 25 months of follow-up.

### Patient 2

A 59-year-old female with a long-standing history of asymptomatic bradycardia presented on 07/01/2024 with a 3-day history of chest pain, presyncope and dyspnoea. Stress testing and echocardiography were unremarkable. A chest pain episode was associated with inferior ST-elevation and frequent premature ventricular contractions. Amlodipine (2.5 mg daily) was initiated for clinical suspicion of intermittent coronary spasm. Both symptomatic bradycardia and Torsade de Pointes were considered in the differential. For this reason, a permanent pacemaker was installed on 12/01/2024. Coronary angiography was normal. Subsequent outpatient device interrogation revealed frequent episodes of polymorphic VT. A history of recurrent chest pain precipitating presyncope was obtained.

She was re-admitted on 27/01/2024. Telemetry revealed recurrent inferior ST-elevation complicated by runs of polymorphic VT. Isordil (10 mg PO TID) and nebivolol (5 mg PO daily) were initiated with no further episodes of chest pain or polymorphic VT before transfer to a tertiary care centre 30/01/2024 for ICRT.

Coronary angiography was unremarkable. Microvascular function was normal. Intracoronary acetylcholine (100ug) induced pan-arterial spasm. The pacemaker was upgraded to an ICD. She was discharged on nebivolol (increased to 10 mg PO BID), amlodipine (increased to 5 mg PO BID), isordil (unchanged dosage), and atorvastatin (40 mg PO QHS). She noted some intermittent, mild chest pain episodes over the next two months without presyncope and then became asymptomatic with no further cardiac events through 05/2025.

### Patient 3

A 50-year-old woman with a history of chronic regional pain syndrome presented on 08/2021 with a resuscitated out-of-hospital cardiac arrest preceded by chest pain that prompted a 911 call just before her collapse. She required multiple cardioversions at the scene (rhythm unreported). Troponin-T-Hs rose to 2053ug/mL. Coronary angiogram and cardiac MRI at a tertiary care centre were reportedly normal. She underwent ICD implantation, and bisoprolol (10 mg daily) was initiated.

She presented 03/2023 with recurrent chest pain associated with palpitations, presyncope, and an ICD shock, then twice during the Summer of 2023 with negative troponins. She was admitted in late 08/2023 following an ICD shock. ICD interrogation revealed a VT that was successfully terminated by defibrillation. Medications remained unchanged.

She was admitted on 09/2023 with chest pain, transient inferolateral ST-elevation, and 102 ug/mL troponin-T-Hs peak. Coronary angiography was normal except for a short myocardial bridge in the mid-distal LAD. She was discharged on amlodipine (2.5 mg daily), bisoprolol (10 mg daily), rosuvastatin (40 mg daily), and acetylsalicylic acid (81 mg PO QAMCC). She was readmitted 2 days later with recurrent chest pain and negative troponins. Diltiazem (30 mg daily) was prescribed.

She was readmitted 10/2023 with recurrent inferior ST-elevation and non-sustained VT with multiple daily episodes. The electrophysiology study failed to initiate VT. Bisoprolol and diltiazem were discontinued. She received 2 doses of carvedilol, then was discharged on nebivolol (10 mg PO daily) and her existing medications. She returned 01/2024 for ICRT. Symptoms had become rare (∼once weekly), short-lived, and mild. Microvascular function was normal. Diffuse acetylcholine(40ug)-induced vasoconstriction (∼50%) was seen in the LAD, not meeting criteria for spasm. Focal spasm of the right coronary artery was noted. Apart from two brief anginal episodes in 03/2024, she has been asymptomatic from a cardiac standpoint through 03/2025.

## Discussion

The pathophysiology of epicardial vasospasm is complex. The salutary response of our patients to nebivolol reflects this complexity and suggests a rethinking of our clinical approach may be helpful. Traditional coronary artery disease risk factors increase the risk of vasospasm, but it can occur in the absence of atherosclerotic disease.^1–3^ Triggers include but are not limited to chronic stress, cold exposure, inflammation, emotional stress, exercise, alcohol withdrawal, tobacco, vasoactive, or stimulant drug use, magnesium deficiency, and hyperventilation.^1–3^

The standard clinical framework explaining epicardial vasospasm emphasizes an imbalance between local vasodilatory signals and VSMC hyperreactivity, the latter being considered preponderant.^1,2^ Accordingly, the standard treatment strategy (CCBs and LANs) aims to increase the delivery of nitric oxide and reduce the contractile tendencies of VSMCs. Despite the potent vasodilatory effects of these medications, randomized controlled trial (RCT) data provide underwhelming evidence of their success. In an RCT of endotypic-guided treatment of patients with angina with non-obstructive coronary arteries, vasospasm diagnosis prompted increased use of CCBs and nitrates and a reduction in beta-blockade.^11^ Under this strategy, symptoms, vasomotor function, and quality of life did not improve, with a statistically insignificant 50% increased risk of major adverse cardiovascular events.^11^ In another RCT, diltiazem improved the results of ICRT, but again without improving patient outcomes or reducing anginal burden (Seattle Angina Questionnaire—80.6 ± 7.9 vs. 82.5 ± 10.4—*P* = 0.39).^12^ The relative inefficacy of these vasodilatory therapies suggests that we may have overestimated the significance of imbalanced local vasodilatory and vasoconstrictory signals in the instigation of vasospasm and that a more holistic framework is required, particularly within the high-risk setting of concomitant ventricular arrhythmias. These concerns are consistent with the observations from this case series in which aggressive vasodilator therapy did not eliminate recurrent spasm or ventricular arrhythmias.

Beta-blockers represent first-line medical therapy for VT and cardiac arrest^6^ but are contraindicated for epicardial vasospasm.^7^ The rationale for the latter is not based on RCT data, but on anecdotal evidence and the rational concern that non-specific beta-blockade, including inhibition of beta-2- and -3-mediated vasodilation, would provoke spasm due to unopposed alpha-mediated vasoconstriction.^7^

Nebivolol is a third-generation cardioselective beta-blocker that minimizes this risk by being highly selective to beta-1 with the highest affinity among current agents.^9^ It is similarly distinct from earlier-generation beta-blockers by promoting endothelial function: Compared to second-generation metoprolol, it reduces endothelin-1-mediated vasoconstrictor tone in adults with elevated blood pressure.^13^ Nebivolol therapy increases nitric oxide release^10^ and brachial artery lumen diameter,^14^ decreases circulating levels of high-sensitivity C-reactive-protein,^14^ and prevents vascular nitric oxide uncoupling,^10^ which would otherwise promote vasoconstriction by increasing oxidative stress. It improved exercise capacity and reduced angina with no adverse events noted in patients with ‘coronary syndrome X’, who have an expected rate of epicardial vasospasm approaching 60%.^15^ In patients with documented epicardial vasospasm, a RCT revealed that while nebivolol was less effective than diltiazem at improving the ICRT response, treatment eliminated the prevalence of >75% of spasm at the time of repeat ICRT after 12 weeks of therapy, and, more importantly, reduced anginal burden (Seattle Angina Questionnaire—81.0 ± 9.2 vs. 86.9 ± 5—*P* = 0.016).^12^ Similarly, the combined nebivolol-diltiazem group performed inferiorly to diltiazem patients in the ICRT, but again improved anginal status (Seattle Angina Questionnaire—79.5 ± 7.9 vs. 85.4 ± 7.8—*P* = 0.002).^12^ Clinically, these studies suggest a significant benefit from nebivolol. Theoretically, the fact that the benefits from nebivolol occurred despite less impressive effects on ICRT imply a complicated relationship between the response to ICRT and clinical outcomes.

It is within this context that this case series should be interpreted. Our patients’ successful clinical courses propose both a potential role for nebivolol in the setting of vasospasm and concomitant VT and a possible pathophysiological explanation. The sympathetic nervous system is hierarchical, with preganglionic spinal cord neurons extending to postganglionic neurons, which branch to respective targets. Its ultimate effects are dependent on both its objective activity and the target’s subjective response to it. Meta-iodobenzylguanidine imaging data propose that both ventricular arrhythmias and coronary spasm are associated with abnormal sympathetic input.^16,17^ This is reinforced by the findings that left stellate ganglion blockade successfully treats refractory coronary spasm,^18^ and decreased sympathetic input with deep sedation and stellate ganglion blockade are effective in the setting of recurrent VT due to vasospasm.^19^ The difference between spasm or VT patients and spasm plus VT patients may therefore be their distributions of sympathetic irregularities. Isolated vasospasm or VT patients may predominantly exhibit localized hyperreactivity to sympathetic inputs, which would both explain their independent manifestations and support spasm treatment with CCBs. Conversely, those who experience both afflictions may be principally or alternatively subject to a hyperactive sympathetic system itself. Thus, nebivolol’s ability to not only diminish the response to sympathetic input, but to indirectly attenuate sympathetic tone,^20^ could explain its efficacy in this particular setting.

This report has obvious limitations, including its observational nature and small sample size. Nevertheless, *Figure 1* chronicles a compelling timeline illustrating the continuation of symptoms throughout CCB and/or LAN use, then their dramatic improvement or resolution following nebivolol commencement. Whether this effect is limited to those who fail conventional therapy or is applicable to vasospasm in general is unknown. Additionally, as each of our patients used nebivolol in conjunction with CCBs, it is unclear whether beneficial effects would be seen with monotherapy or if concurrent vasodilatory therapy is necessary. This latter point is important. None of these patients received nebivolol without concomitant vasodilators, and all had ICDs implanted. This case series does not provide support for the use of nebivolol in the absence of these other measures. Nebivolol’s isolated efficacy should be the focus of future studies.

The first patient did not demonstrate clear STE during MINOCA, but the electrocardiogram changes were consistent with reperfusion events, which may reflect the transient nature of the spasm. The other two showed clear STE during clinical events, and all three had severe vasospasm in response to acetylcholine, meeting the diagnostic criteria.

In summary, this report illuminates the clinically relevant utility of nebivolol in conjunction with other vasodilatory therapies in the management of vasospasm complicated by ventricular arrhythmias. Further, it hypothesizes an important role of the sympathetic nervous system in promoting concomitant vasospasm and VT.

## Lead author biography


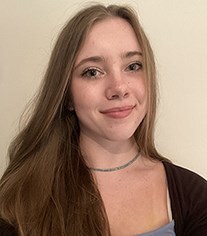


Sarah Miner obtained a Bachelor of Science (BSc) from McGill University in 2024. She is currently a clinical writer at Southlake Regional Health Centre, Newmarket, Canada, and a researcher at the Hospital for Sick Children, Toronto, Canada.

## Supplementary Material

ytaf641_Supplementary_Data

## Data Availability

All data used in this study are not publicly available according to Southlake Regional Health Centre’s policies. Any queries surrounding these article’s data can be sent to the corresponding author’s email.
