# Supplementary material for: Epicardial vasospasm and concomitant ventricular tachycardia treated with Beta-1-specific Beta-blockade: a case series in support of nebivolol
Source: Eur Heart J Case Rep. 2025 Dec 16;10(1):ytaf641. doi: 10.1093/ehjcr/ytaf641 (PMC12836413; doi:10.1093/ehjcr/ytaf641)

**Supplemental Figure 1A:** Patient 1 Normal Electrocardiogram


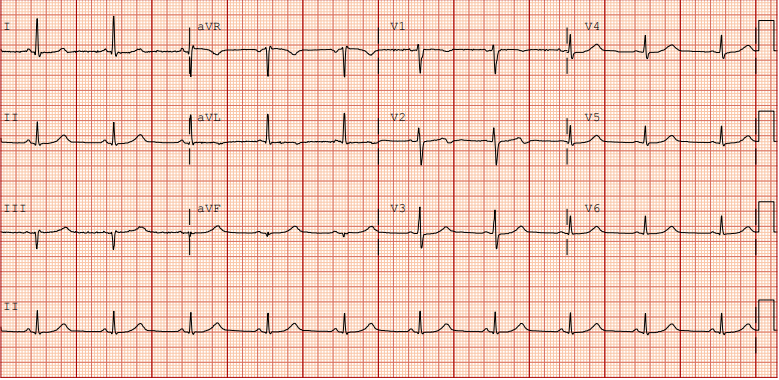


**Supplemental Figure 1B:** Patient 1 Electrocardiogram with Inferior ST-Elevation and Anterior T-Wave Inversions 04/2021


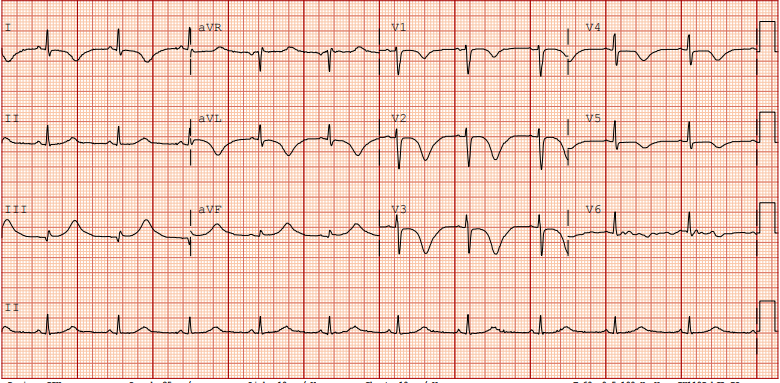


**Supplemental Figure 2A:** Patient 2 Normal Telemetry 09/01/2024


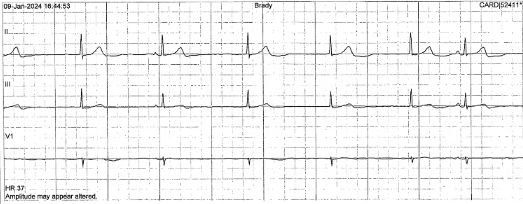


**Supplemental Figure 2B:** Patient 2 Telemetry Showing ST-Elevation with Non-Sustained Polymorphic Ventricular Tachycardia 07/01/2024


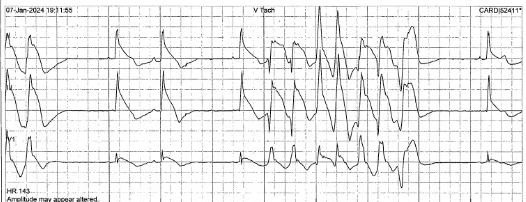


**Supplemental Figure 2C:** Patient 2 Telemetry Revealing Persistent Ventricular Arrhythmia Preceded by ST-Elevation


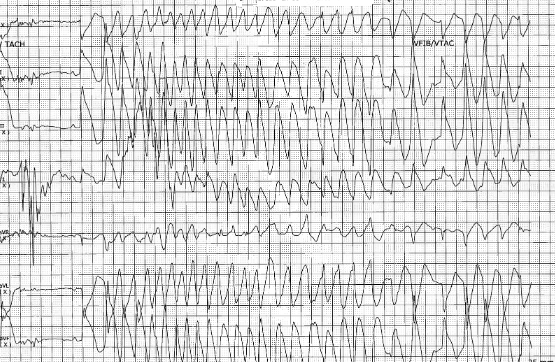


**Supplemental Figure 3A:** Patient 3 Inferior ST-Elevation 05/09/2023


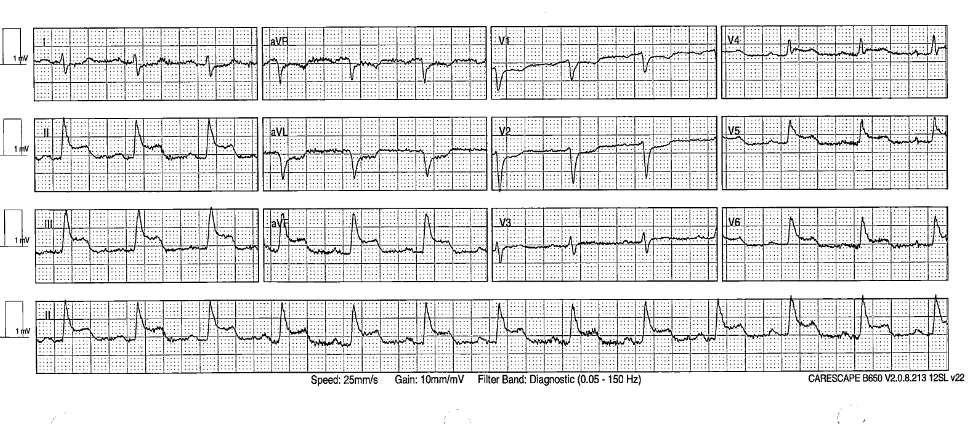


**Supplemental Figure 3B:** Patient 3 Post-Inferior ST-Elevation 05/09/2023


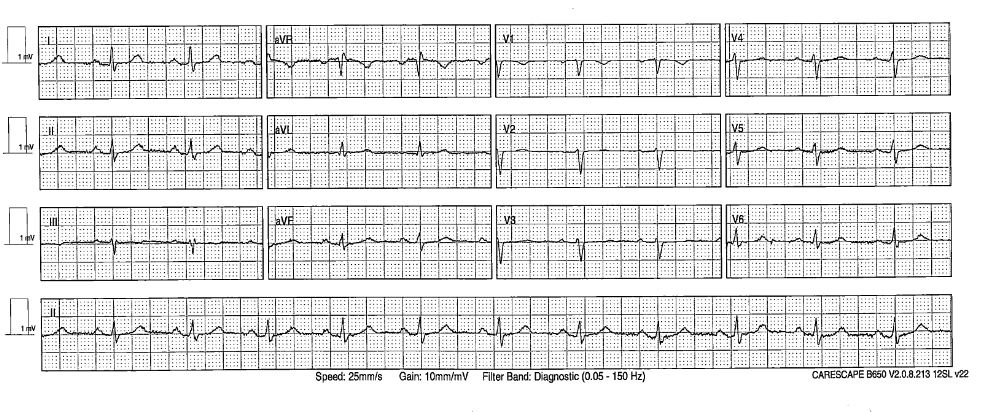

Supplement: ytaf641_Supplementary_Data [file ytaf641_supplementary_data.docx]
